# CBCT location of the fusion between the buccal and lingual cortical in the mandibular ramus: importance to sagittal split osteotomy

**DOI:** 10.4317/medoral.21632

**Published:** 2017-06-18

**Authors:** Leandro Scomparin, Mariana-Quirino-Silveira Soares, Cassia-Maria-Fischer Rubira, Renato-Yassutaka-Faria Yaedú, Thaís-Sumie-Nozu Imada, Bruna-Stuchi Centurion, Elen-Sousa Tolentino, José-Roberto-Pereira Lauris, Izabel-Regina-Fischer Rubira-Bullen

**Affiliations:** 1 Department of Stomatology, Bauru School of Dentistry, University of São Paulo, Bauru, São Paulo - Brazil

## Abstract

**Background:**

Mandibular Sagittal Split Osteotomy (MSSO) is a popular technique in orthognathic surgery used both to advance and to retreat the mandible. However, MSSO may incur in important complications, such as bad splits and sensorineural injuries. Knowing the location of the fusion between the buccal and lingual cortical (FBLC) in the mandibular ramus and the bone thickness in the region where osteotomies will be performed is determinant in MSSO planning to avoid complications. The aim of this study was to document and evaluate possible differences between sexes regarding the location of the FBLC in relation to the superior cortical of mandibular foramen (MF) and bone thickness in the region of interest for MSSO in a Brazilian population.

**Material and Methods:**

Eighty five cone-beam Computed Tomography (CBCT) scans were used to perform linear measurements to determine the location of the FBLC. Bone thickness from the mandibular canal (MC) to the cortical external surfaces and the diameter of the MC were measured at three different points: mandibular ramus (A), mandibular angle (B) and mesial of the second molar (C).

**Results:**

The FBLC was located at a mean distance of 8.3 mm from the superior cortical of the MF in males and 8.1 mm in females. There was no difference between males and females regarding the mean bone thickness from the MC to the buccal external surface at all the points investigated (*p* >>0.05). Bone thickness from the lingual external surface to the MC was bigger among females than males in regions B and C (*p*<0.05). The diameter of the MC was bigger among males in regions B and C.

**Conclusions:**

Sexual dimorphism regarding mandibular bone thickness but not regarding the location of FBLC was present. This fundamental knowledge may assist to the panning of MSSO.

** Key words:**Cone-Beam Computed Tomography, mandibular nerve, orthognathic surgery, sagittal split ramus osteotomy.

## Introduction

Mandibular sagittal split osteotomy (MSSO) is one of the most common surgical procedures used in orthognathic surgery to correct dentofacial skeletal abnormalities. MSSO main indications are related to mandibular retrognathism, prognathism and asymmetry (1). It was first described by Trauner and Obwegeser in 1957 and since then several modifications were purposed focused on decreasing relapse, improving healing and decreasing complications (2). Patients treated with MSSO report a significant improvement in oral and general health related quality of life and psychological function (3). Nevertheless, MSSO can incur in important complications, such as unfavorable fractures (bad splits), permanent sensorineural disturbances and postoperative infections (4).

Since the osteotomy is performed in the vicinity of the inferior alveolar nerve (IAN), the sensorineural injuries are the most common complications (5). The main symptoms of these injures are neurosensory disturbances in the lower lip, gingiva, and chin region, which occur in 20–98% of cases immediately after surgery and in 0–82% of cases during a monitoring period of 6 months to 1 year (6,7).

The occurrence of bad split also represents an important complication and has a reported incidence between 0.5 and 14.6% per MSSO (4). Eventually, bad splits may lead to infections; kidnappings of bone fragments; retardation of bone healing; nonunion, postoperative instability; and dysfunction in the temporomandibular joint. Such complications can also negatively affect the recovery and daily life of patients submitted to orthognathic surgery (8-10).

The presence and positioning of mandibular third molars, surgeon inexperience, osteotomy design and mandibular morphology have been pointed as risk factors related to bad splits (4). There is a great concern in the literature about the fusion of the buccal and lingual mandibular cortical above the mandibular foramen (MF), since bad splits supposedly occur when an osteotomy is performed above or just at this point, at which there is no medullary bone (11-13). Previous investigations have suggested that lesions on the IAN are also more likely to occur in patients who present a thin medullary bone between the mandibular canal (MC) and the external mandibular cortical (14,15). It seems to be clear that the positioning and depth of the sagittal and vertical cuts during the MSSO should be decided based on the bone thickness and mandibular morphology (16).

Hence, the aim of this study is to verify the location of the FBLC regarding the MF, the bone thickness in the region of interest for MSSO and to evaluate possible differences between sexes with cone beam computed tomography (CBCT) in a Brazilian popula-tion.

## Material and Methods

This research was approved by the Ethics Committee on Human Research. This study was carried in 2011 and 2012. CBCT scans performed from 2009 to 2012 with voxel size of 0.3 mm and with acquisition protocol involving the mandible were selected from the archives of the Radiology Department at the Bauru Dental School, Brazil. Men and women who presented facial symmetry were included. Edentulous patients, patients with second molars absence and those with mandibular internal fixation accessories, impacted teeth, anomalies, or neoplasms that might influence the shape of the mandible were excluded from the sample. All images were obtained with the i-CAT Classic (Imaging Science International, Hatfield, Pennsylvania, USA).

Clinical data (age, sex and presence of facial symmetry) were obtained from the patients records.

- Image Analysis

Panoramic and parasagittal reconstructions of CBCT exams were analyzed using i-CAT Vision software to identify the FBLC in the mandibular ramus. Orientation in all three planes of space was carried out before the measurement. The linear measures of the distance of the FBLC from the superior cortical of the MF and of the bone thickness between the MC and the mandibular cortical were performed with the tool “distance”.

**Figure 1:**
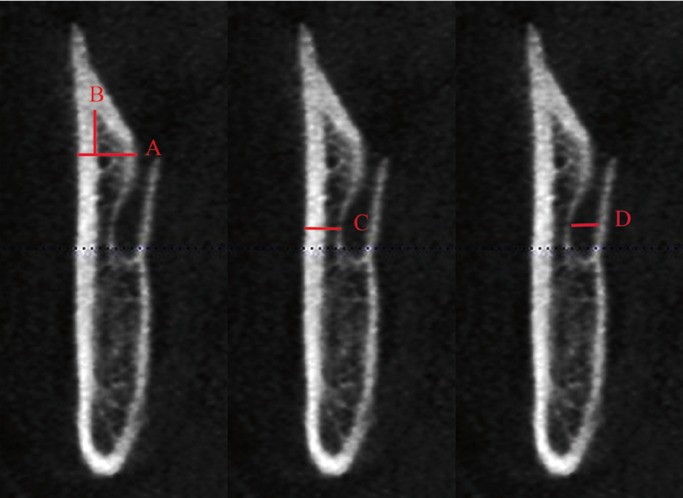
A- line delimiting the upper portion of the MC; B- distance of the fusion of the buccal and lingual cortical from MC; C- MC diameter; D- distance from the cortical bone to the MC.

**Figure 2:**
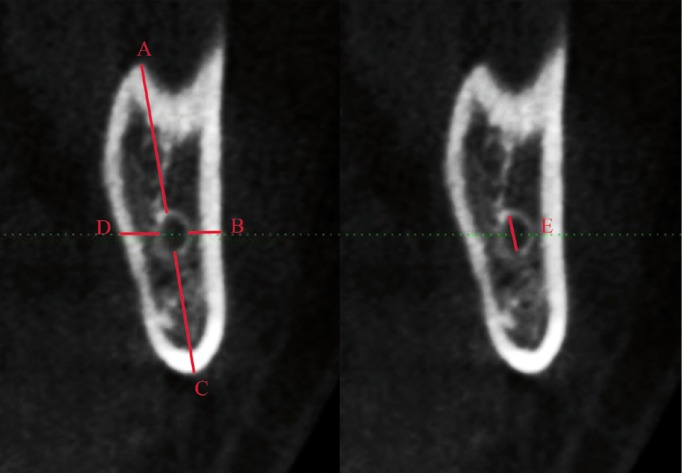
A- distance of the alveolar crest to the MC in the transition region of the body and branch; B- distance from MC to the buccal bone cortical; C- distance from the base jaw to the MC; D- distance to the lingual cortical MC; E- MC diameter.

**Figure 3:**
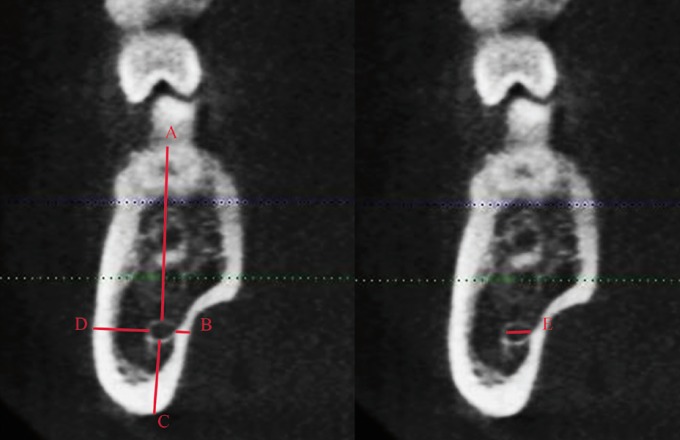
A- distance of the alveolar crest to the MC; B- distance from MC to the buccal bone cortical; C- distance from the base jaw to the MC; D- distance to the lingual cortical MC; E- MC diameter.

For the linear measurement of the distance between the MC and the mandibular cortical, 3 specific regions were predetermined based on the region of interest to MSSO: A) the mandibular nerve entrance in the foramen (determined by the first view in which the foramen is detected in parasagittal reconstructions), B) the region of transition between the ramus and the mandibular body (obtained through a straight line that crosses the MC by tapping the most anterior mandibular ramus), and C) the mesial of the second molar (Figs. 1-3). The largest diameter of the MC was also measured in these 3 regions.

The level of agreement was calculated by k-statistics. To obtain an adequate level of intra-observer agreement 30 images were measured twice with an interval of 30 days.

Statistica for Windows® software (5.1, StatSoft, Tulsa, Oklahoma, USA) was used to analyze the collected data, and the accepted statistical significance was *p* < 0.05. First, Shapiro-Wilk test of normality was performed. Following this procedure, to compare the measurements obtained in the right and left hemi-mandibles and the measurements between sexes Student’s T-test was used for parametric data and Mann-Whitney U test was used for non-parametric data. Continuous data were presented as minimum, maximum and mean ± standard deviation.

## Results

The kappa of intra-observer agreement was 0.87. Eighty five CBCT exams from patients ranging from 15 to 53 years old were evaluated (53 female/32 male), with a mean age of 24.98 years (± 7.95). Among the 170 hemi-mandibles, there were no differences between the measures in the right and left side (*p*>0.05); hence the data from both sides have been presented together. The FBLC was located at an average distance of 8.3 mm ± 2.8 for males and 8.1 mm ± 3.0 for females above the superior cortical of the MF, and no significant difference between sexes was observed (*p* = 0.885).

The average bone thickness from the buccal cortical to the MC in males was 2.8 mm in Region A, 4.0 mm in Region B, and 4.9 mm in Region C. Among females, it was 2.9 mm in Region A, 3.8 mm in Region B, and 4.6 mm in Region C, with no significant statistical difference between them. The bone thickness between the MC and lingual cortical was smaller in males than in females in the Regions B (*p* = 0.006) and C (*p* = 0.001).

Regarding the diameter of the MC, no difference between males and females was observed in Region A (*p* = 0.123). However, males presented a bigger MC diameter than females in Regions B (males 3.9 mm; females 3.5 mm; *p* = 0.005) and C (males 3.0 mm; females 2.7 mm; *p* = 0.043). The bone thickness between the superior cortical and the MC was higher in males than in females (17.2 mm and 16.2 mm, respectively; *p* = 0.019) in Region C, and there was no difference in Region B (*p* = 0.403).

The bone thickness from the mandibular inferior cortical to the MC was significantly higher in males region C (males 7.4 mm; females 6.5 mm; *p* = 0.021). The average minimum and maximum measurements and standard deviation among males and females are presented in  Table 1.

**Table 1:**
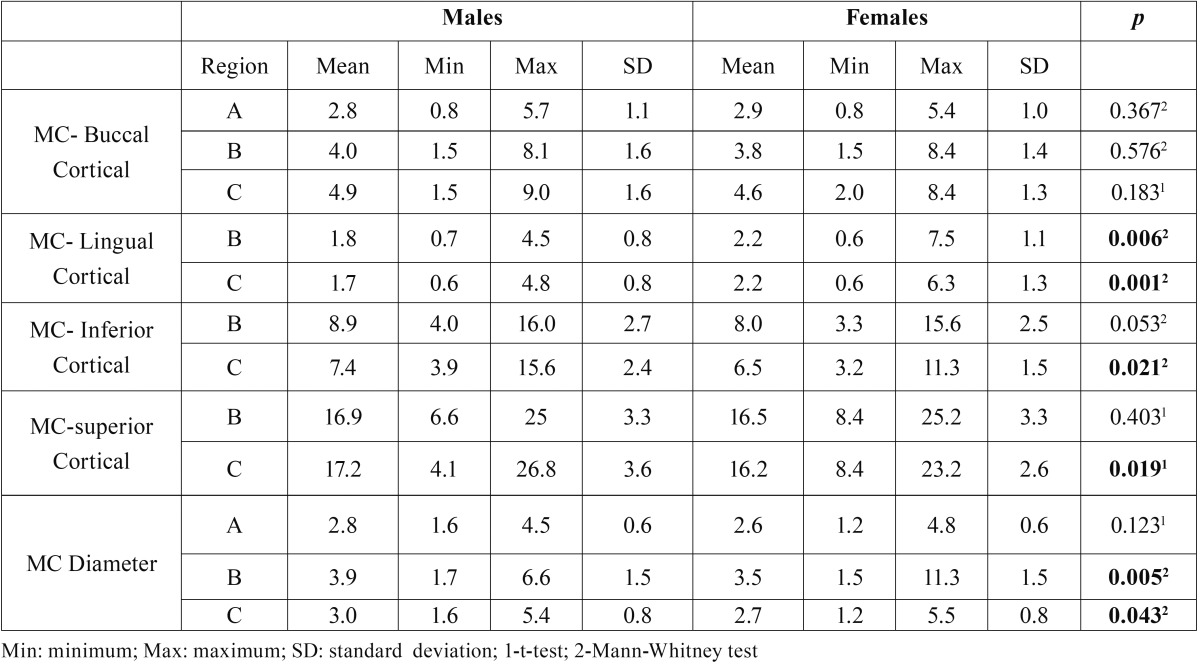
Average measurements, minimum, maximum and standard deviation among males and females in millimeters. 1 Student’s t-test; 2 Mann-Whitney U test; bold values: *p*<0.05).

## Discussion

Patients with dentofacial deformities have been benefited from the technical improvements in MSSO with regard to addressing their aesthetic–functional, psychological, and social issues (17-19). Nevertheless, there are important complications that can compromise the success of this surgical procedure, such as injury to the IAN and the occurrence of bad splits (20-22). The positioning of the medial horizontal osteotomy at the point of the FBLC of the mandibular ramus or higher to this point has been pointed to contribute to the occurrence of unexpected fractures (11). Hence, the knowledge of the FBLC location is important during surgical planning to choose the safest surgical site for osteotomy and the best technique.

In this study, the FBLC was located at a mean height of 8.3 mm in males and 8.1 mm in females to the MF, with no significant difference between sexes. Thus, it is interesting that surgeons avoid performing an internal horizontal mandibular ramus osteotomy at a point above 7 mm from the MF, avoiding a region that could considerably increase the incidence of unfavorable fractures. An investigation of retrognathic and prognathic patients performed by Noleto *et al.* (13) reported that the point of the FBLC was located at 8.95 mm above the lingula for patients with prognathism and 9.41 mm for those with retrognathia. Nevertheless, the authors did not present any data regarding the comparison of the measurements in terms of sex (13). In our study the superior cortical of the MF and not the lingula was used as landmark for the measurements what can difficult the comparison between both studies.

Previous investigations have proposed that the vertical anterior osteotomy is to be performed along the crest of the external oblique line, more precisely between the first and second molars, where presumably, there is a higher buccal bone thickness and a lower chance of IAN injury and badsplits (16,23,24). Our data support this statement, since the biggest distance between the MC and the buccal cortical was observed in this region. The mean bone thickness among the MC and the buccal cortical in this region was 4.9 mm in males and 4.6 mm in females, with no significant difference. Aarabi *et al.* (25) reported that patients with thinner buccolingual distance in the ramus and shorter ramus were more likely to present bad splits in the lingual side of the distal segment during MSSO. A shorter ramus probably presents a smaller distance of the FBLC from the MF that should be considered during MSSO planning. Patients that presented a thinner buccolingual distance in the retromolar area also presented an increased risk for unfavorable fractures in the buccal or lingual side of the distal or proximal segments (25). The authors suggest that the surgical access may be more difficult in smaller mandibles. Furthermore, the smaller amount of bone may contribute to mandible brittleness towards the forces applied during osteotomy (25).

Regarding the vertical dimension of the posterior region of the mandible, our data showed a significant difference in bone thickness between the mandibular inferior and superior cortical to the MC in males and females in Region C, showing that female mandibles have a small vertical diameter than males in this region of interest for the MSSO. In Region C, the thickness between the mandibular inferior cortical and the MC mean was 7.4 mm among males and 6.5 mm among females, a lower measurement than that reported by Yu *et al.* (16), who observed a mean of 7.8 mm ± 1.59 among males and 7.3 mm ± 1.78 among females, with no significant difference between sexes. Witherow *et al.* (26), in a study with panoramic radiographs, reported that patients who presented smaller height of the mandible (less than 2 cm) in the retromolar region and a smaller distance between de apex of the last molar tooth and the inferior cortical of the mandible (less than 0.6 mm) were more likely to present postoperative fractures of the lingual plate. This information should be considered wile planning the surgery (26).

Regarding the bone thickness between the MC and the lingual cortical, the mean was about 2 mm for the regions investigated. These results are close to the findings of De Oliveira *et al.* (27) in a Brazilian population using helical computed-tomography images. The authors reported that in the posterior region, the MC was located near the lingual cortical, with an average distance close to the distance observed our investigation (ranging from 3.80 mm ± 0.82 and 2.10 mm ± 0.62), with its position gradually approaching the buccal cortical during its course to the anterior region (27).

We observed a significantly smaller bone thickness between the MC and the lingual cortical in regions B and C in males when compared to females. These data corroborate the findings of Sekerci and Sahman (28) however; their measurements had been performed at different points in the region of interest for the MSSO. It is important to consider that in patients with thinner lingual plate the use of plates and monocortical screws should be preferred for fixation, since they avoid the transmission of forces to the lingual plate and may reduce de risk of postoperative fractures (26).

In this study, the MC presented a variable diameter that increased in Region B and decreased again in Region C (close to diameter of Region A). In Region C, the mean diameter of the MC was 3.0 mm among males and 2.7 mm among females, which is in agreement with the findings of De Oliveira-Santos *et al.* (29), since the results of the MC diameter ranged between 2.1 and 4 mm in 74% of their research sample.

This research represents a sample of the Brazilian population, which could be the reason for any discrepancies when compared to studies from other countries. In addition, our sample consists of normal patients, which lead us to question whether there would be considerable anatomical differences between these patients and patients with maxillofacial discrepancies. Thus, future studies with other populations and patients with clear indications for MSSO should be encouraged to add evidence to contribute to the prevention of complications of MSSO.

In conclusion, the distance of the FBLC from the MF in the ramus was not influenced by sex and presented a mean length of 8.3 mm for males and 8.1 mm for females. The mean bone thickness and diameter of the MC showed significant sexual dimorphism that should be considered during MSSO planning. Finally, CBCT is an important resource in the surgical planning of MSSO and should guide an individualized treatment plan for each patient.
